# Neuropsychiatric symptoms and severity of dementia

**DOI:** 10.1590/S1980-57642013DN70200006

**Published:** 2013

**Authors:** Gustavo Henrique de Oliveira Caldas, Sueli Luciano Pires, Milton Luiz Gorzoni

**Affiliations:** 1 Resident in Geriatrics Fellowship of Santa Casa de São Paulo (ISCMSP), São Paulo SP, Brazil;; 2 Teaching Professor, Santa Casa de São Paulo Medical School and Technical Director of the Hospital Geriátrico e de Convalescentes Dom Pedro II of ISCSMSP, São Paulo SP, Brazil;; 3 Adjunct Professor and Coordinator of Disciplines of Geriatrics and Gerontology Fundamentals - Santa Casa de São Paulo Medical School, São Paulo SP, Brazil.

**Keywords:** elderly, dementia, neuropsychiatric symptoms, behavioral and psychological symptoms in dementia

## Abstract

Neuropsychiatric symptoms (NPS) cause distress, disabilitiy and reduced quality of life for both the patient and their families

**OBJECTIVE:**

To evaluate the prevalence of NPS as a specific stage of dementia status.

**METHODS:**

A cross-sectional study in patients attending an outpatient clinic for dementia was performed. We applied the Neuropsychiatric Inventory and Clinical Dementia Rating (CDR) scale. Statistical analysis was carried out with SPSS 17 software.

**RESULTS:**

The 124 subjects (mean age of 80.4±7.0 years), 88 women (70.9%) had average duration of dementia of 7.1±3.2 years, most common dementias of Alzheimer's disease (35.5%) and mixed (31.5%) and most prevalent NPS of apathy (75%) and irritability (66.9%). Correlation between apathy and a CDR 1 had a PR (prevalence ratio) = 0.289 and p<0.001while between apathy and CDR 4-5 (PR=8.333, p<0.005). A similar result was found between aberrant motor behavior (AMB) and CDR 1 (PR=0.352, p<0.003) and between AMB and CDR4-5 (PR=2.929, p<0.006).

**CONCLUSION:**

Alzheimer's disease and mixed dementia were predominant, while apathy and AMB were detected in association with the progressive stages of dementia.

## Abstract (pt)

Sintomas neuropsiquiátricos (SNPs) causam sofrimento, incapacidade e redução da qualidade de vida do paciente e das famílias.

**OBJETIVO:**

Avaliar a prevalência de SNPs específicos conforme a fase do estado demencial.

**MÉTODOS:**

Estudo transversal em pacientes atendidos em ambulatório de demências. Aplicou-se Inventário Neuropsiquiátrico e *Clinical Dementia Rating* (CDR). Realizada análise estatística com o software SPSS 17.

**RESULTADOS:**

124 idosos (idade média de 80,4±7,0 anos), 88 mulheres (70,9%). Tempo médio de demência: 7,1±3,2 anos. Demências mais frequentes: doença de Alzheimer (35,5%) e mista (31,5%). Sintomas neuropsiquiátricos mais prevalentes: apatia (75%) e irritabilidade (66,9%). Correlação entre apatia e CDR 1 tem RP (razão de prevalências) = 0,289 e p<0,001 e entre apatia e CDR 4-5; RP=8,333; p<0,005, o mesmo ocorrendo entre comportamento motor aberrante (CMA) e CDR 1 (RP=0,352; p<0.003) e CMA e CDR4-5 (RP=2,929; p<0,006).

**CONCLUSÃO:**

Houve predomínio de demência de Alzheimer e demência mista e se detectou apatia e CMA em associação progressiva com as fases do quadro demencial.

## INTRODUCTION

The elderly population has increased in Brazil since 1960, with the prospect that the country will soon rank sixth worldwide in absolute number of inhabitants over 60 years of age.^1^ This fact implies a rise in degenerative diseases such as dementia.^2^

Dementia is defined as a neuropsychiatric disorder characterized by progressive and acquired deficit in intellectual ability, affecting memory, language, visuospatial skills, cognition, emotion and personality.^3^ It is divided into several subgroups characterized by risk factors, pathophysiological mechanisms, symptoms, neuroimaging, and response to treatment.^6^ Different regions have varying percentages of inhabitants with dementia and subtypes. These discrepancies are justified by these methodologies and socio-cultural and regional standards. The average prevalence found in studies conducted in South America is around 7.1%.^4,5^ Studies conducted from 1994 to 2000 have found prevalence rates ranging from 4.2% in Canada to 14.5% in Spain, while Japan and the U.S.A. have reported prevalence rates of between 5,5% and 9,0% for subjects aged 65 or over.^5^

The main cause of dementia in Brazil is Alzheimer's disease (50%-60% of cases), followed by vascular dementia and mixed dementia(10% to 20% each), frontotemporal dementia (12%) and Lewy body disease (10%15%). Tertiary centers have also observed a prevalence of 0%-37% of potentially reversible dementias.^7-9^

Dementia has, in addition to cognitive impairments, behavioral and psychological symptoms (changes in sensory perception, in thought content, mood and behavior).^10^ These symptoms cause distress, disability, reduced quality of life and increased financial burden for both the patient and their families and have become indicators of early institutionalization.^11,12^ During the evolution of the loss of cognitive status, it is estimated that approximately 80% of patients with Alzheimer's disease have at least one of these symptoms.^7,13,14^

Given these data, it becomes necessary in order to provide local assistance to patients with dementia, to analyze these cases, determine the pattern of neuropsychiatric symptoms (NPS) observed and consequently define strategies to improve the quality of life of patients and their caregivers.

The aim of this study was to evaluate the NPS in patients treated as outpatients for dementia at the Hospital Geriátrico e de Convalescentes Dom Pedro II (HGCDPII) - Irmandade da Santa Casa de Misericórido de São Paulo (ISCMSP).

## METHODS

The dementia outpatient clinic is run at the HGCDPII weekly, with the participation of residents in geriatrics and tutors in the same specialty, all linked to ISCMSP. Being local specialized care, the patients admitted primarily have early and intermediate stages of dementia.

The cases included in this study had, on outpatient admission, DSM IV (Diagnostic and Statistical Manual of Mental Disorders) criteria for dementia and were aged 60 years and over. One of the authors (GHOC) evaluated patients between October 2011 and January 2012, discussing cases with other authors. This study was performed on selected patients who were preferentially elderly with complementary tests available (computed tomography or magnetic resonance imaging of the skull, TSH, glucose, blood count, serum vitamin B12, serum folate, serum creatinine and urea, liver function, electrocardiogram, chest radiography, sodium, potassium, calcium and serological tests for syphilis and HIV).

This was a cross-sectional study using a questionnaire comprising the neuropsychiatric inventory plus a few further questions: date of interview, interview number, patient name, name of caregiver, type of dementia by DSM-IV and stage of dementia based on the Clinical Dementia Rating (CDR) scale. Social and demographic data, gathered from the charts of patients were also analyzed.^5,8,15-17^

The severity of the patient for NPS was measured in three grades, according to the neuropsychiatric Inventory:

1. Mild - produces little distress to the patient.
2. Moderate - more disturbing to the patient but the caregiver can redirect.
3. Severe -very disturbing to the patient and difficult to redirect.

Suffering / caregiver stress and the NPS were divided into five levels, according to the neuropsychiatric Inventory:

1. Minimal Suffering.
2. Mild Distress.
3. Moderate Distress.
4. Severe Distress.
5. Very severe suffering.^8^

Quantitative variables were retained in bivariate analysis using the Chi-square or Fisher's exact test to explore the magnitude of associations (prevalence) between the independent and dependent variables. The dependent variables used were: delusions, hallucinations, agitation, depression, anxiety, euphoria, apathy, lack of inhibition, irritability, aberrant motor behavior, nighttime behaviors, appetite disorders and nutrition. The independent variable was CDR, where CDR 4 and 5 were combined in the same group for statistical purposes, since the clinic served a small number of patients with these degrees of dementia.

This study considered p<0.05as significant and adopted a significance level of 95.0%. Statistical analysis was performed using the Statistical Package for Social Sciences (SPSS) version 17. This study was submitted to and approved by the Ethics in Research ISCMSP Committee (Protocol No. 241/11).

## RESULTS

The sample analyzed consisted of 124 patients, 88 women (70.96%), with mean age of 80.4±7.00 years and mean disease duration of 7.18±3.22 years. A total of 28 (22.6%) patients had CDR 1, 32 (25.8%) CDR 2, 38 (30.7%) CDR 3, 22 (17.7%) CDR 4 and 04 (3.2%) CDR 5.

Types of dementias, 44 patients (35.5%) had Alzheimer's disease, 39 (31.5%) mixed dementia, 23 (18.6%) vascular dementia, seven (5.64%) Lewy body disease, three (2.4%) frontotemporal dementia, three (2.4%) semantic dementia and five patients (4.0%) had potentially reversible dementias.

NPS by frequency of appearance: apathy (75%), irritability (66.9%), nocturnal behaviors (58.8%), depression (57.2%), agitation (56.4%), aberrant motor behavior (53.2%), appetite disorders and nutrition (52.4%), anxiety (50.8%), hallucinations (34.6%), delusions (29.0%), lack of inhibition (21.7%), and euphoria (15.3%).

NPS by severity of patient's suffering in caregiver's opinions: depression (45.1%), apathy (31.2%), anxiety (28.6%), irritability (27.7%), agitation (27.2%), nighttime behaviors (26.0%), aberrant motor behavior (24.3%), appetite disorders and nutrition (20.0%), delusions (16.7%), lack of inhibition (14.8%), hallucination (11.6%) and euphoria (10.5%).

There were 25 caregivers (69.4%) with stress associated with patient delusion, of whom seven (19.4%) had pain grade 5, four (11.1%) grade 4, 11 (30.6%) grade 3 two (5.6%) grade 2 and one (2.7%) grade 1. Prevalence of stress for other NPS: 80.2% (57 caregivers) for depression, 75.7% (53) agitated, 73.0% (46) anxiety, 70.9% (66) apathy, 65, 0% (54) irritability, 60.4% (26) hallucination, 54.5% (36) aberrant motor behavior, 51.8% (14) lack of inhibition, 49.3% (36) nocturnal behavior, 44.6% (29) disorders of appetite and feeding, and 36.8% (7) euphoria. The ranks of the above-mentioned NPS regarding the degree of caregiver stress are shown in Table 1.

**Table 1:** Percentages of different degrees of caregiver stress in subgroups of neuropsychiatric symptoms (NPS) of dementia in a dementia outpatient clinic of the Hospital Geriátrico e de Convalescentes Dom Pedro II, São Paulo (SP).

Symptoms	Grade 0 (No.)	Grade 1 (No.)	Grade 2 (No.)	Grade 3 (No.)	Grade 4 (No.)	Grade 5 (No.)
Delusion	11 (30.6%)	1 (2.7%)	2 (5.6%)	11 (30.6%)	4 (11.1%)	7 (19.4%)
Hallucination	17 (39.5%)	-	2 (4.7%)	21 (48.8%)	1 (2.3%)	2 (4.7%)
Agitation	17 (24.3%)	-	3 (4.3%)	33 (47.1%)	2 (2.9%)	15 (21.4%)
Depression	14 (19.7%)	2 (2.8%)	11 (15.5%)	34 (47.9%)	2 (2.8%)	8 (11.3%)
Anxiety	17 (27%)	4 (6.4%)	6 (9.5%)	29 (46%)	1 (1.6%)	6 (9.5%)
Euphoria	12 (63.1%)	-	-	6 (31.6%)	-	1 (5.3%)
Apathy	27 (29%)	2 (2.2%)	9 (9.7%)	43 (46.2%)	-	12 (12.9%)
Disinhibition	13 (48.2%)	2 (7.4%)	2 (7.4%)	7 (25.9%)	-	3 (11.1%)
Irritability	29 (35%)	2 (2.4%)	4 (4.8%)	32 (38.6%)	6 (7.2%)	10 (12%)
Aberrant motor behavior	30 (45.5%)	-	3 (4.6%)	22 (33.3%)	1 (1.5%)	10 (15.1%)
Nighttime behavior	37 (50.7%)	-	7 (9.6%)	10 (13.7%)	1 (1.4%)	18 (24.6%)
Disorders of appetite and nutrition	36 (55.4%)	1 (1.5%)	4 (6.2%)	15 (23.1%)	3 (4.6%)	6 (9.2%)

No.: number; %: Percentage. Suffering / caregiver stress and the Neuropsychiatric symptoms were divided into five levels: Grade 1: minimal suffering; Grade 2: mild distress; Grade 3: moderate distress; Grade 4: severe distress; Grade 5: very severe suffering.

The prevalence of NPS according to the severity of dementia showed changes in patterns. The correlation between apathy and CDR 1 is protective (PR=0.289, p<0.001), i.e. with low occurrence of this symptom in CDR 1, and risk between apathy and CDR 4-5 (PR= 8.333, p<0.005), i.e. with high occurrence of this symptom in CDR 4-5. The same was found between aberrant motor behavior and CDR 1 (R=0.352, p<0.003) as well as aberrant motor behavior and CDR4-5 (PR=2.929, p<0.006). This study also observed a higher prevalence of anxiety in patients with CDR 1 (PR=2.044, p<0.04) and lower prevalence of hallucination (PR=0.266, p<0.002).

Delusion, lack of inhibition, irritability, and appetite disorders and nutrition had increased prevalence rates up to CDR 3, with subsequent decline in CDR 4-5 but without statistical significance. Irritability approached statistical correlation risk in CDR 3 (PR=1.852, p<0.059).

Depression and nocturnal behaviors did not vary with severity of dementia, suggesting a higher constant prevalence throughout the natural history of dementia, according to the survey data, although there is a known lack of diagnostic criteria for depression in later stages of dementia.^3^

Euphoria, in turn, tended to increase in CDR 3 and decline in CDR 4-5, with statistical significance in CDR 3.

Agitation correlated to protection in CDR 1 (PR= 0.210, p<0.001) and risk in CDR 3 (PR=2.893, p<0.001) with statistical significance, indicating one of the symptoms that worsened during the evolution of dementia. Details of the prevalence ratios and their statistical significance are shown in Table 2, 3, 4, 5.

**Table 2:** Bivariate analysis of change in prevalence of neuropsychiatric symptoms (hyperactivity) according to severity of dementia in a dementia outpatient clinic of the Hospital Geriátrico e de Convalescentes Dom Pedro II, São Paulo (SP).

Crossed variables	PR	[ CI - 95% ]		Value P
		**( - )**	**(+)**	
CDR1 vs agitation	0.210	0.092	0.482	<0.001*
CDR2 vs agitation	0.992	0.544	1.810	0.979
CDR3 vs agitation	2.893	1.445	5.793	<0.001*
CDR4 +5 vs agitation	1.234	0.609	2.500	0.556
CDR1 vs euphoria	0.205	0.030	1.417	0.071
CDR2 vs euphoria	0.368	0.096	1.415	0.153
CDR3 vs euphoria	2.251	1.362	3.722	0.005*
CDR4 +5 vs euphoria	0.781	0.328	1.860	0.568
CDR1 vs lack of inhibition	0.781	0.328	1.860	0.568
CDR2 vs lack of inhibition	0.829	0.381	1.806	0.630
CDR3 vs lack of inhibition	1.464	0.839	2.553	0.198
CDR4 +5 vs lack of inhibition	0.855	0.356	2.056	0.724

CDR: Clinical Dementia Rating; vs: versus; PR: prevalence ratios; CI: confidence interval;

* Significant results.

**Table 3:** Bivariate analysis of change in prevalence of neuropsychiatric symptoms (psychosis) according to severity of dementia in a dementia outpatient clinic of the Hospital Geriátrico e de Convalescentes Dom Pedro II, São Paulo (SP).

Crossed variables	PR	[ CI - 95% ]		Value P
		**( - )**	**(+)**	
CDR1 vs delusions	0.978	0.475	2.014	0.951
CDR2 vs delusions	1.111	0.586	2.105	0.748
CDR3 vs delusions	1.271	0.736	2.196	0.398
CDR4 +5 vs delusions	0.582	0.238	1.424	0.216
CDR1 vs hallucinations	0.226	0.072	0.706	0.002*
CDR2 vs hallucinations	1.130	0.612	2.086	0.697
CDR3 vs hallucinations	1.525	0.905	2.569	0.118
CDR4 +5 vs hallucinations	1.381	0.697	2.739	0.358

CDR: Clinical Dementia Rating; vs: versus; PR: prevalence ratios; CI: confidence interval;

* Significant results.

**Table 4:** Bivariate analysis of change in prevalence of neuropsychiatric symptoms (fourth group) according to severity of dementia in a dementia outpatient clinic of the Hospital Geriátrico e de Convalescentes Dom Pedro II. São Paulo (SP).

Crossed variables	PR	[ CI - 95% ]		Value P
		**( - )**	**(+)**	
CDR1 vs aberrant motor behavior	0.352	0.168	0.737	0.003*
CDR2 vs aberrant motor behavior	0.684	0.374	1.249	0.212
CDR3 vs aberrant motor behavior	1.506	0.863	2.629	0.141
CDR4 +5 vs aberrant motor behavior	2.929	1.263	6.795	0.006*
CDR1 vs appetite disorders	0.659	0.341	1.275	0.211
CDR2 vs appetite disorders	0.996	0.548	1.811	0.989
CDR3 vs appetite disorders	1.208	0.705	2.071	0.489
CDR4 +5 vs appetite disorders	1.198	0.599	2.397	0.608
CDR1 vs nocturnal behaviors	0.932	0.483	1.798	0.833
CDR2 vs nocturnal behaviors	1.021	0.556	1.876	0.946
CDR3 vs nocturnal behaviors	0.863	0.508	1.466	0.587
CDR4 +5 vs nocturnal behaviors	1.320	0.639	2.723	0.448

CDR: Clinical Dementia Rating; vs: versus; PR: prevalence ratios; CI: confidence interval;

* Significant results.

**Table 5:** Bivariate analysis of change in prevalence of neuropsychiatric symptoms (affectiv symptoms) according to severity of dementia in a dementia outpatient clinic of the Hospital Geriátrico e de Convalescentes Dom Pedro II, São Paulo (SP).

Crossed variables	PR	[ CI - 95% ]		Value P
		**( - )**	**(+)**	
CDR1 vs. anxiety	2.044	1.044	4.160	0.040*
CDR2 vs anxiety	0.854	0.470	1.554	0.606
CDR3 vs. anxiety	1.076	0.633	1.829	0.787
CDR4 +5 vs anxiety	0.513	0.248	1.061	0.063
CDR1 vs Apathy	0.289	0.155	0.538	<0.001*
CDR2 vs Apathy	1.000	0.502	1.922	1.000
CDR3 vs Apathy	1.476	0.724	3.010	0.261
CDR4 +5 vs Apathy	8.333	1.177	58.985	0.005*
CDR1 vs depression	0.995	0.515	1.923	0.989
CDR2 vs depression	1.244	0.699	2.315	0.487
CDR3 vs depression	1.145	0.664	1.973	0.625
CDR4 +5 vs depression	0.640	0.323	1.268	0.198
CDR1 vs Irritability	0.659	0.344	1.259	0.211
CDR2 vs Irritability	0.722	0.397	1.313	0.291
CDR3 vs Irritability	1.852	0.934	3.672	0.059
CDR4 +5 vs Irritability	1.111	0.528	2.339	0.780

CDR: Clinical Dementia Rating; vs: versus; PR: prevalence ratios; CI: confidence interval;

* Significant results.

## DISCUSSION

Dementia increases progressively and significantly with advancing age, with rates doubling in the elderly population every five years. There are, however, local variations in prevalence with the majority of studies reporting rates of between 4.2% and 7.2%.^5,9^ Alzheimer's disease accounts for 50%-60% of cases, representing the most common cause of dementia. In our study however, the AD dementia rate of 35.5% together with high prevalence rates for mixed (31.5%) and vascular (18.6%) dementias, may imply a lack of finer control of risk factors for vascular disease, a frequent phenomenon in disadvantaged regions. Moreover, the development of high technology devices providing increasingly sophisticated imaging could be contributing to the increased prevalence of more vascular dementia-related causes.^8,9,18,19^

The low prevalence of frontotemporal dementia (2.4%) in the present study is probably due to the age group assessed, as individuals aged 60 years or older were analyzed whereas this type of dementia typically affects individuals from a younger age group.^20^ The prevalence of potentially reversible dementias (4%) perhaps reflected a lack of cases associated with the use of medications and the conditions of depression, in addition to subject age and the outpatient source.^7,21^

The literature ranks (in descending order of prevalence) apathy, depression, agitation and irritability as the main dementia-related NPS.^7,8^ The present study found these NPS, but in terms of frequency, in a different order, namely: apathy, irritability, nocturnal behaviors and depression, a finding possibly justified by the methodology and/or criteria used and the degree of dementia of the other samples. Some studies have found apathy to be the most frequent Behavioral and Psychological Symptom in Dementia in patients with Alzheimer's disease, while the least common symptom described is euphoria.^13^ There was also another order of decreasing symptoms upon examining the degree of suffering they caused to the patient in caregiver depression, agitation and anxiety.

The suffering of patients according to caregivers revealed that depression, agitation and anxiety are the most reported NPS, although nocturnal behaviors, agitation and delusion, in descending order, caused more severe distress to caregivers. This corresponds with studies where agitation was the NPS most associated with the impact and depression in caregivers along with depression, apathy, lack of appetite and anxiety.^13,20^

Although behavioral and psychological symptoms are ubiquitous in all dementias, their frequency and distribution may vary according to type, severity of dementia and ethnic group.^22^

Studies suggest there is progression in the frequency of some NPSs among patients in the mild stage of dementia (CDR 1) to the more advanced stages (CDR 4-5). The sample analyzed found only apathy and aberrant motor behavior to follow this trend, with statistical significance. Standard criteria for detecting NPS were again employed to justify the data. Depression and nocturnal behaviors were also unchanged in prevalence across the different stages of dementia.

In conclusion, this study found, in the series examined: [1] mixed dementia showed a high prevalence in this population, requiring further studies to better explain this data; [2] apathy, irritability and nocturnal behaviors were observed for the NPS and call for the definition of management protocols for these patients in terms of priority a multidisciplinary approach; [3] depression, agitation and anxiety were the NPSs that caused most distress to caregivers in terms of frequency;

[4] nocturnal behavior, agitation and delirium caused more distress to caregivers in terms of intensity, serving as a warning sign for caregiver stress; and [5] apathy and aberrant motor behavior showed increased frequencies in the later stages of dementia.
